# 525例肺癌中ALK阳性病例临床病理特征研究及检测方法探讨

**DOI:** 10.3779/j.issn.1009-3419.2014.03.08

**Published:** 2014-03-20

**Authors:** 翔 朱, 红威 李, 宝山 曹, 晨 柳, 莉 梁, 玉湘 王, 江峰 由, 菲 高, 晓龙 马, 岩 刘, 华 王, 燕 张, 剑 陈, 波 张

**Affiliations:** 1 100191 北京，北京大学医学部病理学系/北京大学第三医院病理科 Department of Pathology, Peking University, Health Science Center/Peking University Third Hospital, Beijing 100191, China; 2 100022 北京，北京市垂杨柳医院 Department of Pathology, Beijing Chui Yang Liu Hospital, Beijing 100022, China; 3 100191 北京，北京大学第三医院肿瘤化疗与放疗病科 Department of Oncology and Radiation Sickness, Cancer Center, Peking University Third Hospital, Beijing 100191, China; 4 100191 北京，北京大学第三医院放射科 Department of Radiology, Peking University Third Hospital, Beijing 100191, China; 5 100053 北京，基因科技（上海）有限公司 Gene Tech (Shanghai) Company Limited, Beijing 100053, China

**Keywords:** 肺肿瘤, ALK, EGFR, 免疫组化, FISH, Lung neoplasms, Anaplastic lymphoma kinase gene (ALK), Epidermal growth factor receptor (EGFR), Immunohistochemistry (IHC), Fluorescence in situ hybridization (FISH)

## Abstract

**背景与目的:**

间变性淋巴瘤激酶（anaplastic lymphoma kinase, *ALK*）融合基因的产生对肺癌的发生、发展起重要作用。ALK酪氨酸激酶抑制剂（tyrosine kinase inhibitors, TKIs）对ALK阳性肺癌患者具有较好的疗效。提高ALK阳性病例的检出率对患者有重要意义。本研究旨在探讨ALK阳性病例的临床病理特征、免疫组化（immunohistochemistry, IHC）方法在筛查ALK阳性病例中的意义，以及荧光原位杂交（fluorescence *in situ* hybridization, FISH）在检测流程中的作用。

**方法:**

应用IHC方法检测525例肺癌患者组织标本中ALK的表达，并对其中34例进行FISH验证。

**结果:**

肺癌病例中ALK IHC阳性率为5.14%（27/525）。ALK阳性组中年轻患者、女性患者比例高于ALK阴性组（*P* < 0.05）；实体型肺腺癌在ALK阳性组中比例明显增高，而腺泡型与贴壁型比例明显降低，与阴性组相比有差异（*P* < 0.05）。34例病例进行FISH验证，IHC与FISH符合率随IHC阳性程度的增加递增。伴或不伴有*EGFR*突变的ALK IHC阳性病例，都必须进行ALK FISH验证。

**结论:**

IHC可以作为可靠的ALK筛查手段，提高ALK的检出率。FISH检测对确诊ALK阳性肺癌具有重要意义。

## Abstract (en)

**Background and objective:**

The fusion (rearrangement) of anaplastic lymphoma kinase (*ALK*) gene has been identified as an import factor to the tumorigenesis and development of lung cancer. ALK tyrosine kinase inhibitors (ALK-TKIs) have been proved to have good effects to ALK positive lung cancers. The increasement of the relevance ratio of ALK will be very important to patients. The aim of this study is to investigate the clinical pathological features of ALK positive lung cancer, and the roles of immunohistochemistry (IHC) and fluorescence in situ hybridization (FISH) in screening and confirming the ALK positive cases in the testing flow of ALK.

**Methods:**

IHC analysis of ALK in tumor specimens was performed on 525 lung cancer patients. 34 cases among them were confirmed by FISH.

**Results:**

The positive incidence of ALK by IHC was 5.14% (27/525). The ALK positive patients were significantly younger than ALK negative patients (*P* < 0.05), and femal was predominant (*P* < 0.05). The proportion of solid predominant adenocarcinoma was significantly higher in ALK positive patients (*P* < 0.05). While acinar and lepidic predominant adenocarcinoma were significantly lower in ALK positive patients (*P* < 0.05). FISH was applied in 34 cases. The coincidence rate was increased with the increasement of positive intensity of IHC staining. All the IHC positive cases with or without *EGFR* mutation must be confirmed by FISH.

**Conclusion:**

IHC is a reliable detection method to screening the ALK in lung cancer, and then enhance the relevance ration. To make a definite diagnosis of ALK positive lung cancer, FISH is a significant detection method.

肺癌是世界上死亡率最高的恶性肿瘤之一^[1]^。近年来，随着其发病率的节节攀升，以分子靶向治疗为核心内容的个体化治疗已经成为肺癌治疗的研究热点，肺癌相关靶向分子的检测也越来越受到关注。棘皮动物微管相关蛋白-间变性淋巴瘤激酶（echinoderm microtubule-associated protein-like 4 gene and the anaplastic lymphoma kinase gene, *EML4-ALK*）融合基因是2007年Soda等^[2]^在非小细胞肺癌（non-small cell lung cancer, NSCLC）患者的肿瘤标本中首次发现。*EML4-ALK*融合基因是由*ALK*基因在2号染色体短臂内倒位inv（2）（p21p23）与其相邻的*EML4*基因重接形成的。有研究^[3]^表明EML4-ALK可诱导肺癌发生，在给予ALK酪氨酸激酶抑制剂（tyrosine kinase inhibitor, TKI）后肿瘤可迅速消退。克唑替尼（crizotinib）作为一种ALK-TKI，对存在*EML4-ALK*融合基因的肺癌患者进行靶向治疗，已正式应用于临床。已有研究^[4]^证实是否存在*EML4-ALK*融合基因对于克唑替尼的疗效密切相关。因此，对肺癌患者的*EML4-ALK*融合基因检测具有十分重要的意义^[5]^。2013年，我国出台了关于中国NSCLC ALK检测的相关指南^[6]^，因此，我们按照指南对实际工作中的病例进行*ALK*基因检测和分析总结，为深入研究我国肺癌病例中*ALK*融合基因状况及阳性病例的临床病理特征，从而为临床进一步的生物靶向治疗提供可靠的依据。

表皮生长因子受体（epidermal growth factor receptor, *EGFR*）基因是与肺癌发生、发展密切相关的另一个重要的驱动基因。人们曾经认为*EGFR*的突变与*ALK*融合基因在同一个病例中是互相排斥的^[7]^，但随着ALK检测的大范围开展，人们发现了*ALK*与*EGFR*双突变的病例^[8, 9]^，临床上这些双突变的患者对EGFR-TKI及ALK-TKI均可能有疗效^[8]^。在我们的病例中这种情况的发生率有多少，免疫组化在确诊双突变中的意义，是我们关注的问题。

## 1 对象与方法

### 1.1 研究对象

选取2012年9月-2013年12月北京大学第三医院病理科的肺癌存档标本及部分来自外院的肺癌会诊病例共525例。肺癌的分型诊断参照世界卫生组织（World Health Organization）编写的肺、胸膜、胸腺及心脏肿瘤病理学和遗传学（2004年版）诊断标准。其中肺腺癌组织学亚型的诊断参照2011年国际肺癌研究协会/美国胸科学会/欧洲呼吸学会（International Association for the Study of Lung Cancer/American Thoracic Society/European Respiratory Society, IASLC/ATS/ERS）联合制定的肺腺癌新分类标准进行分型^[10, 11]^。标本类型包括肺叶切除标本、肺楔形切除标本、CT引导经皮肺穿刺标本、支气管镜粘膜活检标本、锁骨上/腋窝淋巴结活检标本、胸膜活检标本、椎体肿物穿刺以及胸水沉渣。每例标本的病理组织学诊断均由两位有经验的病理医生重新复核。

### 1.2 免疫组化方法检测ALK蛋白的表达

#### 1.2.1 实验方法

525例肺癌组织标本经10%中性福尔马林固定后，常规石蜡包埋，4 μm厚度切片。免疫组化采用Envision法。兔单克隆抗体ALK（D5F3）抗体购自Cell Signaling Technology公司。免疫组化二抗SP检测试剂盒购于北京中杉金桥生物技术有限公司，ALK（D5F3）抗体按1:250稀释），应用PBS代替一抗作为阴性对照，按照试剂说明书进行操作。

#### 1.2.2 结果判读^[6, 12, 13]^

0/阴性：肿瘤细胞明确无着色；1+： > 5%肿瘤细胞呈现微弱或模糊的胞浆着色；2+： > 5%肿瘤细胞中等强度胞浆着色；3+： > 5%肿瘤细胞呈现颗粒状胞浆强着色。

### 1.3 *ALK*基因的FISH检测

#### 1.3.1 实验方法

525例肺癌病例中有34例肺癌标本进行了*ALK*基因的FISH检测，使用经FDA认证的ALK双色分离探针试剂盒（Vysis LSI ALK Dual Color, Break Apart Rearrangement Probe, Abbott Molecular）。根据试剂盒说明进行操作。石蜡切片通过脱蜡等预处理过程之后，依次进行抗原修复、组织消化、变性、孵育杂交、洗涤及核复染。

#### 1.3.2 结果判读

在荧光显微镜下随机选取50个肿瘤细胞，要求肿瘤细胞核轮廓及信号清晰，每个细胞核内至少有一组红、绿信号，如果红绿信号分离（距离≥两个信号直径）或额外出现单独的红色信号，视为阳性细胞，50个观察细胞中阳性细胞 < 5个为阴性标本， > 25个为阳性标本，如果在5个-25个之间则再计数50个细胞，100个细胞内合计阳性细胞数≥15个为阳性标本。

### 1.4 *EGFR*基因检测

525例肺癌病例中有34例肺癌标本应用直接测序法或扩增阻滞突变系统（amplification refractory mutationsystem, ARMS）（Qiagen Inc, Valencia, CA）^[14]^方法检测*EGFR*基因突变情况。实验按照试剂说明书进行操作。

### 1.5 统计学分析

研究数据采用SPSS 19.0进行分析处理。组间率的比较采用*χ*^2^检验。以*P* < 0.05为差异有统计学意义。

## 2 结果

### 2.1 肺癌患者临床资料特点（表 1）

525例肺癌中≥50岁者共476例， < 50岁者共49例，发病中位年龄为63岁（30岁-88岁）。。应用免疫组化方法检测阳性组的患者更年轻，与阴性组相比具有差异（*P*=0.002）。525例肺癌患者中男284例，女241例，男:女=1.169:1。阳性组病例中女性患者更多见，与阴性组相比具有差异（*P*=0.002）。

**1:** 525例肺癌患者肿瘤细胞中ALK蛋白表达情况及与临床病理特征的关系 Correlation between ALK protein expression and clinicopathological factors in 525 lung cancer patients with non-small cell lung cancer

Features	All	ALK protein expression (IHC by using D5F3)		
	(*n*=525)	Positive	Negative	*P*
		(*n*=27)	(*n*=498)	
Age (years)				0.002^*^
≥50	476 (90.67%)	19 (70.37%)	457 (91.77%)	
< 50	49 (9.33%)	8 (29.36%)	41 (8.23%)	
Sex				0.002^*^
Male	284 (54.10%)	7 (25.93%)	277 (55.62%)	
Female	241 (45.90%)	20 (74.07%)	221 (44.38%)	
Histology				
Adenocarcinoma	400 (76.20%)	24 (88.89%)	376 (75.50%)	
Solid predominant	73 (13.90%)	10 (37.04%)	63 (12.65%)	0.002^*^
Acinar predominant	232 (44.19%)	7 (25.93%)	225 (45.18%)	0.037^*^
Papillary predominant	57 (10.86%)	6 (22.22%)	51 (10.24%)	0.061
Micropapillary pre	9 (1.71%)	1 (3.70%)	8 (1.61%)	0.381
Lepidic predominant	29 (5.52%)	0	29 (5.82%)	< 0.001^*^
Squamous cell carcinoma	94 (17.90%)	2 (7.41%)	92 (18.47%)	0.108
Adenosquamous carcinoma	13 (2.48%)	1 (3.70%)	12 (2.41%)	0.501
LCNEC	12 (2.29%)	0	12 (2.41%)	
Mixed small cell/large cell	2 (0.38%)	0	2 (0.40%)	
Carcinoid tumor	2 (0.38%)	0	2 (0.40%)	
Sarcomatoid Ca.	1 (0.19%)	0	1 (0.20%)	
Adenoid cystic Ca.	1 (0.19%)	0	1 (0.20%)	
Other specific histologic features				
Ad. with Signet cells > 10%	7 (1.33%)	1 (3.70%)	6 (1.20%)	0.310
Ad. with mucinous Ad. Diff.	19 (3.62%)	3 (11.11%)	16 (3.21%)	0.068
Ca.: carcinoma; LCNEC: large cell neuroendocrine carcinoma; Ad.: adenocarcinoma; Diff.: differentiation. Statistical analyses were performed using *Pearson Chi-Square* test. ^*^P < 0.05.				

### 2.2 标本类型特点

本次研究标本类型呈多样化特点，最常见的标本类型是CT引导经皮肺穿刺（368例），此外包括较常见的手术切除肺标本（69例）、支气管镜粘膜活检标本（49例）、胸水沉渣（17例）、胸膜活检（8例），以及远处转移灶标本：锁骨上/腋窝淋巴结活检标本（10例）、椎体肿物穿刺（4例）。

### 2.3 标本组织学类型特点（表 1）

525例肺癌经免疫组织化学方法检测ALK（D5F3）在肿瘤细胞胞浆中的表达，阳性者27例，阳性率为5.14%。阳性病例中24例为肺腺癌（阳性率6%），2例为鳞状细胞癌（阳性率2.13%），1例为腺鳞癌（阳性率7.69%）。其余类型的肺癌，如大细胞神经内分泌癌、混合型癌、类癌等肺神经内分泌肿瘤均为阴性。肉瘤样癌及腺样囊性癌均为1例，均阴性。ALK阳性组与ALK阴性组在腺癌的组织学亚型上存在统计学差异（*P* < 0.05）（表 1）。ALK阳性组实体型腺癌更常见（*P*=0.002），而ALK阴性组腺泡型更常见（*P*=0.037）。29例贴壁型肺腺癌ALK免疫组化均为阴性（*P* < 0.001）。乳头型与微乳头型在ALK阳性组与阴性组之间无差异。文献中提到的*ALK*融合基因更常见于印戒细胞 > 10%或伴有粘液腺癌分化的组织学特点^[15]^在本研究中未能体现（*P* > 0.05）。鳞癌及腺鳞癌的比例在ALK阳性组与ALK阴性组之间没有统计学差异（*P* > 0.05）。

### 2.4 525例肺癌患者中34例患者同时进行了ALK FISH检测及EGFR检测的结果

34例患者中14例ALK（D5F3）为阳性，经FISH验证后，其中10例FISH亦为阳性。其中ALK IHC及FISH均阳性的病例多为腺癌，其亚型可为实体型、腺泡型及乳头型（图 1）。34例患者按照*EGFR*的基因状态划分为*EGFR*野生型组及EGFR突变组，分别比较IHC阳性与FISH阳性的符合率（表 2）。*EGFR*野生型组IHC 3+及IHC 1+与FISH阳性的吻合率均为100%，IHC 2+与FISH阳性吻合率为66.67%。*EGFR*突变组IHC 3+与FISH阳性吻合率为50%，IHC 1+与FISH阳性的吻合率为0。两组患者合计后，IHC 3+与FISH阳性吻合率为88.71%，IHC 2+与FISH阳性吻合率为66.67%，IHC 1+与FISH阳性吻合率为50%。无论*EGFR*的基因状态抑或两组合计，IHC阴性与FISH阴性的吻合率均为100%。上述结果所表现出的趋势与文献相符^[16]^。

**1:**
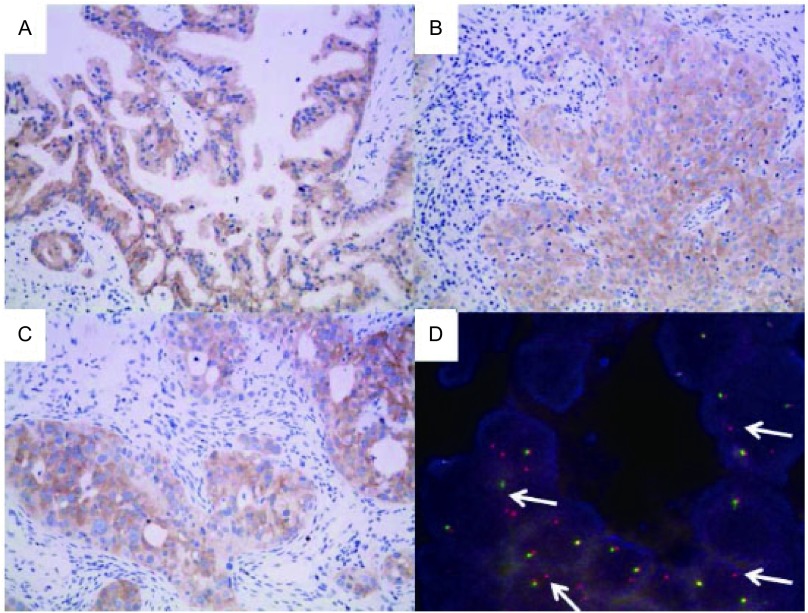
ALK在不同亚型肺腺癌中的表达情况及FISH验证。A：ALK在乳头型肺腺癌肿瘤细胞胞浆内高表达（×200）；B：ALK在实体型肺腺癌肿瘤细胞胞浆内高表达（×200）；C：ALK在腺泡型（筛状型）肺腺癌肿瘤细胞胞浆内高表达（×200）；D：ALK IHC阳性腺泡型肺腺癌FISH检测示肿瘤细胞核内红、绿信号分离或出现额外的红色信号（箭头示），提示*ALK*基因断裂或重组，即阳性。 Expression of ALK in 4 *EGFR* mutated lung adenocarcinomas with different histologic subtypes and the confirmation by FISH. A: High expression of ALK in cytoplasm of papillary predominant adenocarcinoma (×200); B: High expression of ALK in cytoplasm of solid predominant adenocarcinoma (×200); C: High expression of ALK in cytoplasm of acinar (cribriform) predominant adenocarcinoma (×200); D: In ALK IHC positive acinar predominant adenocarcinoma, the results of a break-apart FISH assay of tumor cells with separation of red and green probe signals or extra red probe signals (arrows) indicates a break of ALK or maybe chromosomal rearrangement involving ALK. The FISH result is positive.

**2:** 不同EGFR状态的34例肺癌病例中ALK免疫组化与FISH结果的比较 Comparison between ALK IHC and FISH results in 34 patients with lung cancer and different EGFR status

	Cases	FISH	IHC 3+	IHC 2+	IHC 1+	IHC 0
*EGFR* wild-type	22	Positive	5	2	2	0
		Negative	0	1	0	12
		Coincidence rate	100%	66.67%	100%	100%
*EGFR* mutated	12	Positive	1	0	0	0
		Negative	1	0	2	8
		Coincidence rate	50%	/	0%	100%
Total	34	Positive	6	2	2	0
		Negative	1	1	2	20
		Coincidence rate	85.71%	66.67%	50%	100%

上述34例患者中有4例肺癌患者免疫组化ALK（D5F3）为阳性（图 2），2例为3+，另2例为1+，均为腺泡型腺癌。他们同时存在*EGFR*突变，包括3例19外显子缺失突变，1例21外显子L858R突变（表 3）。对此4例ALK IHC患者进行ALK FISH验证，仅有1例阳性，为34岁男性，ALK IHC是蛋白表达为3+，FISH检测示红、绿信号分离的细胞数超过了15%，达41%（图 2），同时存在EGFR 19外显子的缺失。该患者已服用ALK-TKI，近期效果不错。另外3例无论ALK IHC的表达水平为3+或1+，其ALK FISH验证均为阴性（图 2）。

**2:**
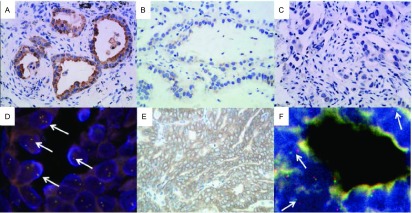
4例*EGFR*突变型肺腺癌中ALK蛋白的表达情况及FISH检测情况。A：第1例腺泡型腺癌（EGFR 21外显子点突变L858R），肿瘤细胞胞浆内ALK蛋白表达为3+（×400）；B：第2例腺泡型腺癌（EGFR 19外显子缺失突变），肿瘤细胞胞浆内ALK蛋白表达为1+（×400）；C：第3例腺泡型腺癌（EGFR 19外显子缺失突变），肿瘤细胞胞浆内ALK蛋白表达为1+（×400）；D：FISH检测示第一例腺癌肿瘤细胞核内分别标记3’端及5’端的红、绿信号相近或融合（箭头示），提示*ALK*基因未发生断裂，为ALK野生型，即阴性；E：第4例腺泡型（筛状）腺癌，（EGFR 19外显子缺失突变），肿瘤细胞胞浆内ALK蛋白表达为3+（×400）；F：FISH检测示第4例腺癌肿瘤细胞核内红、绿信号分离（箭头示），提示*ALK*基因断裂或重组，即阳性。 Expression of ALK protein and FISH detection in 4 *EGFR* mutated lung adenocarcinoma cases. A: High expression (3+) in the first case of acinar predominant lung adenocarcinoma with EGFR exon 21 l858R (×400); B: Low expression (1+) in the second case of acinar predominant lung adenocarcinoma with EGFR exon 19 deletion (×400); C: Low expression (1+) in the third acinar predominant lung adenocarcinoma with EGFR exon 19 deletion (×400); D: The results of a break-apart FISH assay of tumor cells from the first patient with close apposition or confusion of red and green probe signals (arrows) which hybridizes to the 3 region and 5 region of *ALK* gene seperately. It indicates and intact wild-type ALK without broken. The FISH result is negative; E: High expression (3+) in the fourth acinar (cribriform) predominant lung adenocarcinoma with EGFR exon 19 deletion (×400); F: The results of a break-apart FISH assay of tumor cells from the fourth patient with separation of red and green probe signals (arrows) indicates a break of ALK or maybe chromosomal rearrangement involving ALK. The FISH result is positive.

**3:** 4例*EGFR*突变肺腺癌患者的临床病理特征及ALKIHC及FISH检测结果 IHC and FISH detection of ALK in 4 lung adenocarcinoma patients with *EGFR* mutations

Case	Age	Sex	Tissue type	Histology diagnosis	ALK IHC	ALK FISH (rate of positive cells)	EGFR status^*^
1	57	F	Needle biopsy	Ad. Acinar predomiant	3+	Neg. (0%)	Exon 21 L858R
2	63	F	Needle biopsy	Ad. Acinar predomiant	1+	Neg. (0%)	Exon 19 deletion
3	57	F	Needle biopsy	Ad. Acinar predomiant	1+	Neg. (0%)	Exon 19 deletion
4	34	M	Surgical biopsy	Ad. Acinar (cribriform) predomiant	3+	Pos. (41%)	Exon 19 deletion
F: Female; M: Male; Neg.: Negative; Pos.: Positive. ^*^: The detection of EGFR of case 1, case 2 and case 3 by ARMS, case 4 by sequencing.							

## 3 讨论

FISH是研究检测*ALK*融合基因最早也是最公认的方法^[16]^。通过荧光显微镜下的观察，可以原位观测到细胞核内*ALK*基因的断裂，间接提示*ALK*基因可能断裂后与其他基因（如最常见的*EML4*，以及少见的*TFG*、*KIF5B*）相融合，异常激活下游的酪氨酸激酶，促进肺癌的发生发展^[17]^。由于用于FISH检测的探针较为昂贵、荧光显微镜的特殊设备需求、暗视野的读片以及FISH结果的判读，可能使得FISH不易在我国病理科得到广泛普及。随着研究的发展，人们发现D5F3、5A4等免疫组化抗体在肺癌的ALK蛋白表达的检测中具有高度的敏感性和特异性^[18]^，由于经济、易操作在我国病理科开始得到推广。虽然FISH及免疫组化不能明确*ALK*融合基因的类型，但分别从基因及蛋白水平反映了ALK的断裂和表达，是检测ALK阳性肺癌的重要手段。

2013年6月中华病理学杂志发表了吴一龙教授及临床、病理各行业专家共同制定的《中国间变性淋巴瘤激酶（ALK）阳性非小细胞肺癌诊断专家共识（2013版）》（后文简称《共识》）^[6]^，为我们的实际工作操作提供了指南。本研究应用常规免疫组化方法，使用ALK（D5F3）抗体对525例肺癌进行筛查，结果表明阳性患者倾向于年轻女性，与文献^[19]^报道相符。525例肺癌包括了肺腺癌、鳞癌、腺鳞癌及除小细胞癌外的神经内分泌癌等等，总体阳性率为5.14%，与文献报道相符。本研究发现ALK阳性的肺腺癌中分化差的实体型比ALK阴性组更常见，分化较好的腺泡型及贴壁型少见，此与文献^[15]^报道相符。在文献中提到的伴 > 10%印戒细胞及伴粘液腺癌分化的特殊组织学亚型，在本研究的ALK阳性组与阴性组之间未显示出明显差异，可能与病例数较少有关。从另一个方面而言，不能根据组织学亚型的特点进行筛选患者。本研究在鳞癌及腺鳞癌病例中亦有阳性病例检出。上述研究结果提示ALK（D5F3）免疫组化可以作为一个可靠的筛查手段，其筛查的结果、分型的趋势与文献报道相符。本研究标本类型以经皮肺穿刺等小标本为主，并包括胸水沉渣等细胞学标本，结果表明ALK（D5F3）免疫组化方法可以广泛应用于各类标本，结果可靠；并能尽可能地减少漏诊，提高ALK阳性病例的检出率。

《共识》指出常规免疫组化无论阳性信号强弱如何，均应通过FISH、RT-PCTR或Ventana免疫组化进行最终验证。本研究将525例中的34例肺癌标本进行了FISH检测，并对免疫组化的阳性程度与FISH的符合率进行了研究，结果表明IHC阴性者FSIH均为阴性，符合率达100%；免疫组化阳性程度越高，FISH阳性的几率越大；但无论阳性程度如何，均有FISH阴性，即免疫组化假阳性的可能。上述结果与文献^[16]^相符。

*EGFR*在我国NSCLC中的突变率明显高于高加索人，可达40%以上（亚洲人群腺癌突变率为51.4%）^[20]^。本研究中的34例患者同时进行了ALK FISH检测及EGFR检测。过去人们认为*ALK*融合基因与EGFR在NSCLC中是相互排斥的^[7]^，但近年来随着EGFR及ALK检测的推广、检测人群的增加，发现了*EGFR*与*ALK*双突变的人群，并且发现这些患者可能会对EGFR-TKI或ALK-TKI有不同程度的疗效^[8, 9]^。对于病理科而言，EGFR与ALK的检测对指导临床靶向治疗具有重要意义。一般情况患者EGFR检测结果为阴性（野生型）时，再进行ALK检测，即所谓的通过EGFR状态富集ALK检测人群。但是由于*EGFR*与*ALK*双突变的存在，应用免疫组化的方法筛查ALK阳性病例可以防止漏掉阳性的患者，为患者的靶向治疗提供更多的选择机会，并且该方法更加经济、快捷。由于应用ALK（D5F3）免疫组化进行ALK筛查得到结果的速度比EGFR测序或ARMS法检测要快，如果ALK免疫组化为阳性，患者可能会疑问是否还需要进行EGFR检测。我们的研究表明是需要的，理由之一是*EGFR*、*ALK*双突变的存在，另一个理由是ALK免疫组化阳性必须进行FISH或其他方法的验证方可以认为是确切的阳性。

本研究中有4例患者是ALK免疫组化阳性伴有*EGFR*突变，经过FISH验证只有1例EGFR患者ALK FISH阳性，是确切的*EGFR*、*ALK*双突变患者。另外3例*EGFR*突变患者无论ALK免疫组化阳性着染的强度如何，ALK FISH检测均为阴性。该结果提示FISH检测在诊断ALK阳性肺癌及*EGFR*、*ALK*双突变肺癌中具有非常重要的作用。

综上所述，本研究应用免疫组化的方法对525例肺癌*ALK*基因的蛋白表达进行了检测，发现在年轻、女性、实体型腺癌中ALK的表达更为常见。同时发现了*EGFR*突变与*ALK*基因融合共同存在的病例。本研究结果提示了ALK（D5F3）免疫组化方法是一个很好的ALK筛查手段，阳性病例进行ALK FISH验证是保证ALK阳性肺癌诊断的重要环节。
